# Identification and Characterization of a Novel Mannanase from *Klebsiella grimontii*

**DOI:** 10.3390/bioengineering10101230

**Published:** 2023-10-21

**Authors:** Changzheng Chen, Kuikui Li, Tang Li, Junyan Li, Qishun Liu, Heng Yin

**Affiliations:** 1 Dalian Engineering Research Center for Carbohydrate Agricultural Preparations, Dalian Technology Innovation Center for Green Agriculture, Liaoning Provincial Key Laboratory of Carbohydrates, Dalian Institute of Chemical Physics, Chinese Academy of Sciences, Dalian 116023, China; 2 University of Chinese Academy of Sciences, Beijing 100190, China; 3 Key Laboratory of Se-enriched Products Development and Quality Control, Ministry of Agriculture and Rural Affairs, National-Local Joint Engineering Laboratory of Se-enriched Food Development, Ankang 725000, China

**Keywords:** β-Mannanase, Konjac glucomannan, Konjac glucomannan oligosaccharides, *Klebsiella grimontii*, substrate specificity

## Abstract

Konjac glucomannan (KGM) is a natural polysaccharide derived from konjac, which has been widely used in various fields due to its numerous beneficial properties. However, the high viscosity and water absorption of KGM limit its application. Compared with KGM, Konjac glucomannan oligosaccharides (KGMOS) have higher water solubility and stronger application value. In this paper, a novel mannanase *Kg*ManA was cloned from *Klebsiella grimontii* to develop a new KGMOS-producing enzyme. Bioinformatic analysis shows that the structural similarity between *Kg*ManA and other enzymes was less than 18.33%. Phylogenetic analysis shows that *Kg*ManA shares different branches with the traditional mannanases containing the CMB35 domain, indicating that it is a novel mannanase. Then, the enzymatic properties were determined and substrate specificity was characterized. Surprisingly, *Kg*ManA is stable in a very wide pH range of 3.0 to 10.0; it has a special substrate specificity and seems to be active only for mannans without galactose in the side chain. Additionally, the three-dimensional structure of the enzyme was simulated and molecular docking of the mannotetraose substrate was performed. As far as we know, this is the first report to characterize the enzymatic properties and to simulate the structure of mannanase from *K. grimontii*. This work will contribute to the development and characterization of novel *K. grimontii*-derived mannanases. The above results indicate that *Kg*ManA is a promising tool for the production of KGMOS.

## 1. Introduction

Konjac glucomannan (KGM) is a water-soluble dietary fiber derived from konjac tubers [1]. As a high-molecular-weight neutral polysaccharide, KGM is composed of β-D-glucose and β-D-mannose (ratio of 1:1.6) connected by β-1,4-glycosidic bonds, with acetyl groups contained in each 9–19 sugar unit [2]. Due to its abundant resources, biocompatibility, renewability, and excellent film-forming properties, this polysaccharide has received extensive attention [3]. Nowadays, KGM has been utilized for producing food gels, food preservatives, food thickeners, edible and biodegradable packaging films, biomedical materials and health-promoting functional foods [1,3,4,5,6]. However, such a polysaccharide exhibits considerable swelling and viscosity due to its enormous molecular weight and ease of forming hydrophilic colloids in water [7,8]. Additionally, KGM has poor mechanical qualities and could be easily disintegrated under high-humidity conditions [9]. These characteristics prevent KGM from forming films and exhibiting biological activity, which limits its potential use in the production of food and other commodities.

Konjac glucomannan oligosaccharides (KGMOS) are oligosaccharides produced by the breakdown of KGM. KGMOS has a lower molecular weight compared to KGM and contains more acetyl groups and branched chains [10]. A previous study has shown that the presence of the acetyl group in KGMOS makes it a low-calorie addition and cryoprotectant when used to freeze and store seafood [11]. In addition, KGMOS shows lower swelling than KGM, which leads to improved effects in treating constipation [12]. KGMOS also demonstrated a stronger probiotic effect compared to natural KGM. This could result in an increased quantity of probiotics, the inhibition of pathogenic bacteria growth, the promotion of short-chain fatty acids (SCFAs) production, the restoration of function, antioxidant properties, and immune regulation [13,14,15]. Therefore, KGMOS may overcome a number of the shortcomings of KGM. KGMOS is an excellent alternative to KGM and could be better utilized in a wider range of fields [16].

Developing appropriate strategies to degrade KGM is an effective way to increase the value of its products. Currently, chemical hydrolysis (alkaline hydrolysis, acidic hydrolysis, and oxidative hydrolysis), physical degradation (heating, ultrasound, and irradiation), and enzymatic hydrolysis are the most efficient methods for degrading KGM [17]. However, physical degradation consumes a significant amount of energy and results in a wide molecular weight distribution of the hydrolysate. This is due to the combined action of numerous degradation processes, making it difficult to control the conditions of chemical hydrolysis [18]. Furthermore, the environmental pollution, time-consuming nature, substrate browning, and high cost of these strategies make them unsuitable for the large-scale degradation of KGM [19]. Compared to other methods, enzymatic hydrolysis offers several advantages, including high selectivity, effectiveness, safety, and operability. It is currently the most commonly employed strategy for degrading KGM [7]. 

Appropriate carbohydrate-degrading enzymes determine the effectiveness of the KGM enzymatic hydrolysis strategy. One of the effective enzymes for degrading KGM is endo-β-1,4-mannanase (E.C.3.2.1.78), which depolymerizes mannan by initiating the hydrolysis of β-1,4-glycosidic bonds in the polysaccharide backbone [20]. Such β-mannanases can be synthesized by bacteria such as *Thermobifida fusca*, and fungi such as *Aspergillus niger*, *Paenibacillus lentus*, *Bacillus subtilis*, and *Trichoderma longibrachiatum* [21,22,23]. Based on the similarity of amino acid sequences, endo-β-1,4-mannanase is mainly divided into the GH5 and GH26 families, and a few members belong to the GH113 and GH134 families [24]. Endo-β-1,4-mannanases from GH5, GH26, and GH113 belong to the clan GH-A [25]. They all assemble into a similar (β/α)_8_-barrel fold and function through a retention reaction mechanism [26,27,28]. Moreover, endo-β-1,4-mannanase has conserved amino acids that act as an acid/base and a nucleophile at the active site. Mannanases typically have a modular structure composed of two domains. These domains are structurally distinct, and the carbohydrate-binding module (CBM) is the most important non-catalytic module, which enhances the binding of enzymes to insoluble polysaccharide and increases the glycoside hydrolytic efficiency [20].

Endo-β-1,4-mannanases are widely distributed in nature, and bacteria are one of the predominant sources commonly used for their isolation [29]. Most bacterial-derived mannanases have the ability to degrade a variety of natural plant polysaccharides [30]. However, when mixed polysaccharides are used as substrates, they tend to produce oligosaccharides with different branches. Therefore, an enzyme with high specificity for producing a homogeneous substrate is required. This will lower the substrate costs and increase the added value of products. In addition, the tolerance of mannanase was also taken into consideration. Xu et al. enhanced the stability of a mannanase under acidic conditions to improve its application in the food and feed industry [31]. Another report indicated that a novel mannan, *Dt*ManB, derived from *Dictyoglomus thermophilum*, could hydrolyze guar gum at 80 °C, making it particularly suitable for oil drilling [32].

Therefore, based on the objective of developing new KGM-degrading enzymes, a strain *Klebsiella grimontii* DICP B2-5 capable of degrading KGM was isolated in this study. From the genome of this strain, the mannanase *Kg*ManA gene was cloned and expressed heterologously in *Escherichia coli* BL21 (DE3). The activity of the purified enzyme was determined. The substrate specificity, biological properties, and product analysis of *Kg*ManA were characterized. The results show that *Kg*ManA, an enzyme with high specificity for KGM and good pH tolerance, could degrade KGM into oligosaccharides with different degrees of polymerization. In addition, a phylogenetic analysis was performed, and the structure of KgManA was predicted. KgManA has low structural homology (less than 18.33%) with other structurally determined proteins, and is only similar to the putative endo-mannanase in sequence, indicating the structural novelty of *Kg*ManA. The results of the characterization and structural simulation of mannanases derived from *K. grimontii* are reported for the first time, indicating their potential usefulness in development and application.

## 2. Materials and Methods

### 2.1. Materials and Chemicals

*K. grimontii* DICP B2-5 was isolated from rotten konjac and soil in the Ankang konjac planting area. From the Guangzhou RuiBio Company in Guangzhou (China), KGM was purchased. Shanghai Keyuan Industrial Company in Shanghai (China) provided the locust bean gum, fenugreek gum, guar gum, and sesbania gum. From the Takeda-kirin Company in Shanghai (China), Curdlan was purchased. Zymosan was purchased from Sangon Biotech company in Shanghai (China). We bought microcrystalline cellulose and carboxymethyl cellulose from the Sigma Company in Shanghai (China). We bought ivory nut mannan from the Megazyme Company in Beijing (China). All molecular biology supplies, unless otherwise noted, came from the Thermo Fisher Scientific Company in Beijing (China). Synthetic primers along with sequencing services were provided by the Beijing Genomics Institute in Beijing (China).

### 2.2. Expression and Purification of Beta-Endo-Mannanase Gene KgManA from K. grimontii

In this study, the strain *K. grimontii* DICP B2-5, which can degrade KGM, was isolated by repeated screening. The putative endo-1,4-mannanase (called *Kg*ManA) was screened using the existing data of *K. grimontii* in the National Centre for Biotechnology Information (NCBI) and the results of multiple sequence alignment for mannanase. According to the sequence of *Kg*ManA and the restriction sites *Nde*I and *Xho*I in pET21 (+), the primers for cloning were designed in the following way: a forward primer *Kg*ManA-F with a restriction *Nde*I site (underline added) 5′-GATATACATATGGCGGAGCAGTCGCACTTTGAAC-3′ and a reverse primer *Kg*ManA-R with a restriction *Xho*I site (underline added) 5′-GTATAACTCGAGCTCTGCAACCACTTCAATCG-3′. Then the entire gene of *Kg*ManA was amplified from the genomic DNA of *K. grimontii* DICP B2-5 by PCR using primer pairs *Kg*ManA-F/R. The amplified product was ligated to the *Nde*I and *Xho*I restriction site of the vector pET21 (+) by double digestion. An N-terminal (His)_6_-tagged frame was included with the recombinant plasmid pET21a/*Kg*ManA, which was then transferred into *E. coli* BL21 (DE3). Incubating the recombinant strain at 16 °C overnight in a 180 rpm shaker after adding isopropyl-β-D-thiogalactoside (IPTG) of 0.1 mM led to the expression of the *Kg*ManA strain. Centrifugation was used to collect the cells, and a temperature of 4 °C, with 30 replications of 300 W for 3 s on and 5 s off, was used to ultrasonically destroy these cells. The supernatant after centrifuging was then loaded onto an NTA-Ni Sepharose resin (purchased from GE Healthcare company, Beijing, China) of 10 mL, which had been pre-equilibrated with binding solution (20 mM PBS in pH 6.0 with 50 mM NaCl and 20 mM imidazole) in order to purify the (His)6-tagged recombinant *Kg*ManA. In total, 5-column volume (CV) binding solution was used to wash the protein. Then, a 3 CV wash solution (20 mM PBS of pH 6.0 with 50 mM NaCl and 80 mM imidazole) and a 3 CV elution solution (20 mM PBS of pH 60 with 50 mM NaCl and 250 mM imidazole) were used to wash *Kg*ManA and clean the NTA-Ni Sepharose resin. All protein solutions of *Kg*ManA were then analyzed by sodium dodecyl sulfate polyacrylamide gel electrophoresis (SDS-PAGE). The BCA Protein Concentration Assay (purchased from Beyotime Biotechnology company in Shanghai, China) was used to determining the concentration of the crude and purified enzymes with bovine serum albumin as a standard.

### 2.3. Activity Assay of KgManA

The 3,5-dinitrosalicylic acid (DNS) chromogenic method was used to spectrophotometrically (540 nm) determine the mannanase activity of *Kg*ManA, and the calibration curves of mannose were applied as a standard. The quantity of enzyme that catalyzes 1 µmol of hydrolysate per milligram in one minute was defined as one unit of enzyme activity.

The following substrates were examined in order to evaluate the specificity activity of *Kg*ManA among various polysaccharides: KGM, β-1,4-D-mannan, carboxymethyl cellulose (CMC), curdlan, locust bean gum, fenugreek gum, guar gum, Sesbania gum, zymosan A and tara gum. The above polysaccharides were used as substrates (0.5%, *w*/*v*) in the standard reaction, which was carried out in 20 mM PBS buffer (pH 6.0). The mixture was then incubated at 40 °C for 10 min after the addition of *Kg*ManA, and the reaction was stopped by 10 min of boiling in a water bath. Due to the activity of *Kg*ManA against CMC, curdlan, locust bean gum, guar gum, Sesbania gum, zymosan A and tara gum not being detected, the statistical analysis of other polysaccharides’ data was performed only using Prism 9. The Shapiro–Wilk test was conducted, and the statistical results of ordinary one-way ANOVA are presented as an asterisk.

### 2.4. Biochemical Properties of KgManA

By incubating the enzyme with KGM dissolved in different buffers (Glycine-HCl buffer (adjusted to the pH value of 3.0), sodium acetate buffer (pH value adjusted to 4.0 and 5.0), phosphate buffer (pH value adjusted to 6.0 and 7.0), Tris-HCl buffer (pH value of 8.0) and Glycine-NaOH buffer (pH value of 9.0 and 10.0)) under standard assay conditions at 40 °C, the effects of pH on the activity of *Kg*ManA were tested. By incubating the enzyme with KGM at 4, 20, 30, 35, 40, 45, 50 and 60 °C under the optimal pH conditions, the effects of temperature on the activity of *Kg*ManA were tested.

After incubating the enzyme at different pH values at 4 °C for 24 h in buffers with different pH values (listed above), the pH stability of *Kg*ManA was evaluated. The thermostability of *Kg*ManA was evaluated after incubating with *Kg*ManA for 1 h at 25, 30, 35, 40, 45 and 50 °C.

The kinetic parameters were measured using KGM as the substrate; 3 mg/mL, 4 mg/mL, 5 mg/mL, 6 mg/mL, and 7 mg/mL were selected as the appropriate KGM reaction concentrations, and 15 min was selected as the appropriate reaction time. Under these conditions, the reaction of *Kg*ManA with KGM was a one-dimensional kinetic reaction. The enzyme activity of *Kg*ManA was tested by the DNS chromogenic method, and the enzyme concentration was then determined. Then, nine groups of the reciprocal of enzyme-specific activity values (1/v) and the reciprocal of their corresponding KGM concentrations (1/s) were calculated. Using GraphPad Prism 9, the Lineweaver–Burk plot was completed, and the slope value, intercept value, and 95% confidence intervals were obtained. The Michaelis–Menten constant (K_m_) and the rate of reaction (V_max_) were obtained by calculating the slope-to-intercept ratio and the reciprocal of the intercept. By dividing the V_max_ value by the concentration of *Kg*ManA, the catalytic constant (K_cat_) was obtained. Finally, the K_cat_/K_m_ value was calculated. In addition, the 95% confidence interval of all kinetic constants were calculated. 

### 2.5. Hydrolysis Product Analysis of KgManA

After 24 h of digestion, the degraded products of KGM were analyzed using matrix-assisted laser desorption/ionization–time of flight mass spectrometry (MALDI-TOF–MS). The hydrolysis products of the KgManA degradation were tested using an AB SCIEX MALDI-TOF/TOF 5800 profile for mass spectrometry. 

### 2.6. Homology Modeling and Analysis of the Degradation Mechanism

The homology modeling of *Kg*ManA was generated by AlphaFold2 [33]. To explore the degradation mechanism of *Kg*ManA, we docked the N-terminal catalytic domain module of *Kg*ManA with mannotetrose using the Tripos Sybyl X-2.1.1 software package. The model of mannotetrose used for the docking study was downloaded from the GLYCAM database: https://glycam.org/cb/ (accessed on 26 July 2023). The key residues in the catalytic domain and the interaction with the substrate were analyzed.

## 3. Results

### 3.1. Cloning, Expression and Purification of KgManA

From the KGM-degrading bacteria *K. grimontii* DICP B2-5, the *Kg*ManA gene was cloned. The open reading frame (ORF) of *Kg*ManA is composed of 2151 base pairs (bp) (see Supplementary Material Figure S1) with a molecular weight of 79.79 kilodaltons (kDa) (see Supplementary Material Figure S2), encoding 716 amino acids. According to ExPASy’s predicted data, *Kg*ManA has an instability index (II) of 29.95, an *E. coli* half-life of 10 h, a fat index of 69.03, and an average hydrophilic index of −0.517. Therefore, *Kg*ManA could be stably expressed in *E. coli* as a water-soluble protein.

**Figure 1:**
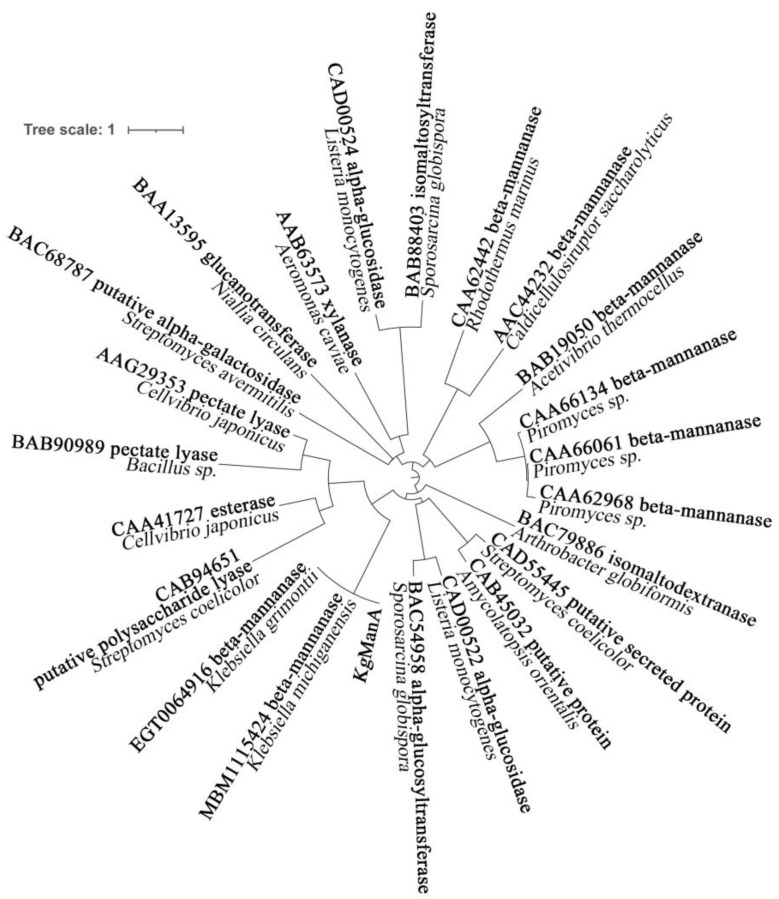
Phylogenetic analysis of *Kg*ManA and other proteins containing CBM35. To establish the phylogenetic tree, all sequences were searched for within and obtained from NCBI. MEGA 11 was used for multiple sequence alignment. PhyloSuite and IQtree were used for sequence cutting and phylogenetic tree construction. The labels of each branch are displayed with Genbank accession number, protein name and derived strain. The sequences of *Kg*ManA are shown in Supplementary Material S3.

The BLASTp results show that *Kg*ManA shared 99.57% similarity with β-1,4-mannanase from *K. grimontii.* The sequence alignment and structure prediction revealed that *Kg*ManA is a multi-domain protein comprising an N-terminal GH5 catalytic domain module, a C-terminal CBM35-binding module, and two intermediate functional domains of unknown function (Fibronectin type 3 domain and myxosortase-dependent M36 family metallopeptidase domain). Since only the CBM35 sequence of KgManA could be aligned in NCBI, the phylogenetic analysis of various proteins with CBM35 is displayed in Figure 1. It should be noted that mannanases derived from *Klebsiella* sp., which exhibit high similarity to *Kg*ManA, do not originate from the same branch as other endo-mannanases.

**Figure 2:**
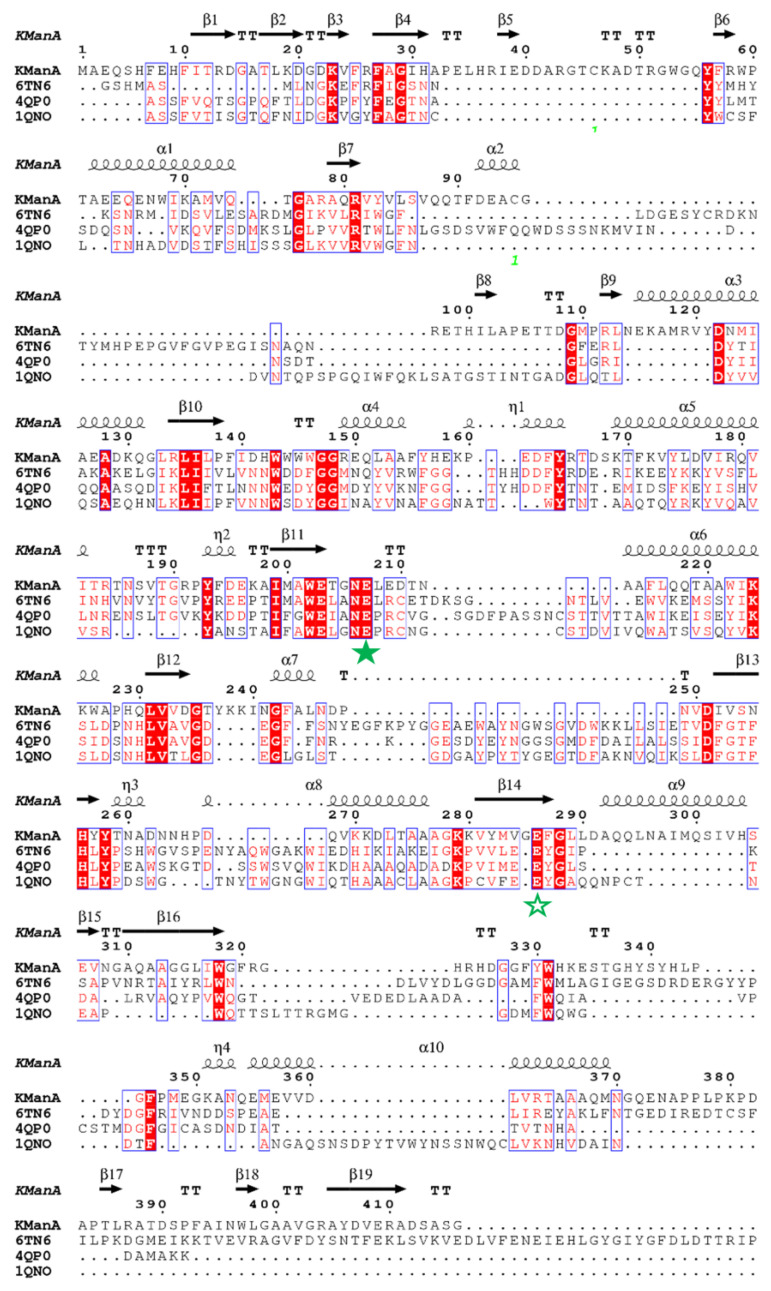
Multiple sequence alignments of *Kg*ManA and related mannanases using ClustalX2 and ESPript 3.0. The sequence alignment was performed using the enzymes’ molecules, similarly to *Kg*ManA, namely, KManA, the sequence of *Kg*ManA, mannanase from *K. grimontii* DICP B2-5; 6TN6, endo-1,4-beta-mannosidase from *T. petrophila*; 4QP0, endo-beta-mannanase from *R. miehei*; 1QNO, endo-beta-mannanase (sequence homology of 14.58%, PDB entry 1QNO) from *T. reesei*. The catalytic residues are indicated by filled (acid/base) and empty (catalytic nucleophile) green stars. The residues that formed the disulphide bridge are indicated by green '1'.

Moreover, the full *Kg*ManA sequence shares low amino acid sequence identity with other structurally determined proteins. Only the N-terminus of *Kg*ManA could retrieve proteins with similar sequences, including endo-1,4-beta-mannosidase (sequence homology of 18.33%, PDB entry 6TN6) from *Thermotoga petrophila* RKU-1, endo-beta-mannanase (sequence homology of 15.19%, PDB entry 4QP0) from *Rhizomucor miehei,* and endo-beta-mannanase (sequence homology of 14.58%, PDB entry 1QNO) from *Trichoderma reesei*. According to the three high-homology characterized proteins (6TN6, 4QP0 and 1QNO), the alignment analysis with the *Kg*ManA N-terminal catalytic module are shown in Figure 2. According to sequence alignment, *Kg*ManA contains conserved residues such as Arg81, Asn206, Glu207, His 256, Tyr258, and Glu286 (Figure 1), which are essential for the catalytic activity of the GH5 family enzyme [34]. Among them, Glu207 and Glu286 act as the catalytic acid base and the nucleophile in the catalytic reaction, respectively.

The coding gene of *Kg*ManA was cloned into the vector pET-21 (+) and expressed heterologously in *E. coli* BL21 (DE3). Subsequently, the recombinant *Kg*ManA was purified using Ni-NTA agarose affinity chromatography and analyzed by SDS-PAGE (see Supplementary Material Figure S2). The results show that recombinant *Kg*ManA could be effectively expressed in *E. coli*, as shown in Figure 2. A single band, ranging from 95 kDa to 72 kDa, can be clearly observed in lane 5. The purification results are listed in Table 1. The specific activity of *Kg*ManA against KGM was 0.22 U/mg, the purification factor was 1.08, and the yield was 0.46%. 

Surprisingly, the catalytic effect of *Kg*ManA on KGM was not strong, and the specific activity of the crude enzyme was only 0.22 U/mg. Moreover, after purification, the specific activity of this enzyme increased by only 8.33%.

### 3.2. Substrate Specificity of KgManA

The substrate specificity of *Kg*ManA was investigated for nine different polysaccharides. As shown in Table 2, *Kg*ManA had a specialized activity toward β-1,4-D-mannan, but only limited activity toward KGM and fenugreek gum (0.43 ± 0.04 U/mg and 0.04 ± 0.01 U/mg), and was essentially weak in relation to other substrates. The results of the substrate specificity test show that *Kg*ManA could degrade glucomannan and mannan, but had no apparent effect on cellulose or galactomannan. This indicates that *Kg*ManA functions as a mannanase.

It was found that *Kg*ManA had the highest hydrolysis activity for 1,4-D mannan, while the enzyme exhibited a catalytic activity of approximately 34.40% for KGM hydrolysis. According to these data, *Kg*ManA primarily breaks down the mannose-containing KGM’s main chain by acting as a mannanase, and it may also generate oligosaccharides with acetyl groups in the side chain. Due to the significant application value of this oligosaccharide product, we will investigate the potential of *Kg*ManA as an enzyme for hydrolyzing KGM. Our objective is to explore its application value and determine whether it exhibits any distinctive hydrolysis characteristics when KGM is used as a substrate.

### 3.3. Biochemical Properties of KgManA

**Figure 3:**
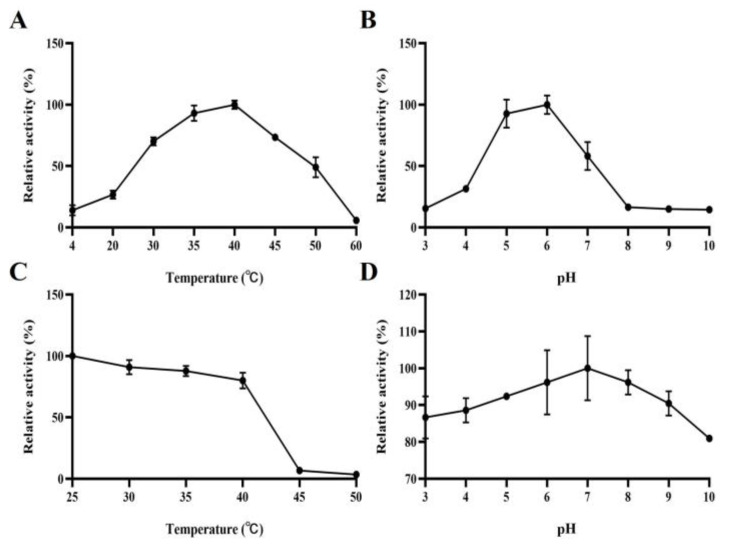
Enzymatic properties of *Kg*ManA. (**A**) The optimal temperature of *Kg*ManA. Activity at 40 °C was taken as 100%. (**B**) The optimal pH of *Kg*ManA. Activity at pH 6.0 was taken as 100%. (**C**) The thermostability of *Kg*ManA. The thermostability was measured at various temperatures between 25 °C and 50 °C. (**D**) The pH stability of *Kg*ManA. The pH stability was measured at different pH (ranging from 3.0 to 10.0) for 24 h at 4 °C. Each value in the data set represents the average of three replicate measurements, with the standard deviation included for accuracy.

When KGM was used as a substrate, the optimum temperature of *Kg*ManA was 40 °C (Figure 3A). According to the investigation of thermostability, *Kg*ManA was found to be sensitive to high temperatures, as it could maintain 80% of its initial activity below 40 °C, and above this temperature, *Kg*ManA basically had no hydrolytic activity for KGM (Figure 3C). This result shows that *Kg*ManA is unsuitable for degrading KGM at high temperatures, and cannot sustain acceptable enzyme activity under hyperthermic conditions.

*Kg*ManA had the highest activity, retaining more than 80% of its maximal activity, at pH 5.0–6.0 (Figure 3B), and after incubation at 4 °C for 24 h, *Kg*ManA showed extreme stability under a wide range of pH (>80% activity was maintained in all measured pH ranges), thus exhibiting high pH stability (Figure 3D). This indicates that *Kg*ManA is able to effectively degrade KGM over a wide pH range. Thus, in order to effectively degrade KGM and produce high-value-added products, a strategy that combines acid/alkali cracking with *Kg*ManA enzymatic hydrolysis may be beneficial.

Through the Lineweaver–Burke plot, the enzyme kinetic parameters of *Kg*ManA were determined. The results show that the intercept in the fitted linear equation is 1.30 and the slope is 11.75. Additionally, when KGM was used as the substrate, *Kg*ManA had a K_m_ value of 9.04 ± 1.11 mg/mL, a V_max_ value of 0.77 ± 0.18 μmol/(min·mg), a K_cat_ value of 1.53 ± 0.36 min^−1^, and a K_cat_/K_m_ value of 0.17 ± 0.03 mg^−1^·mL^−1^·min^−1^ (Table 3).

### 3.4. Analysis of Hydrolysis Products of KgManA

**Figure 4:**
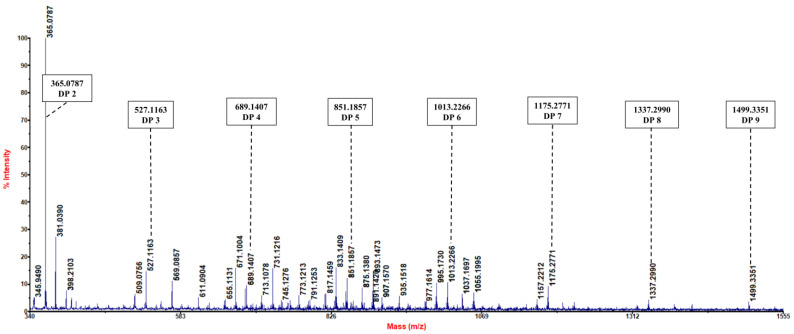
Hydrolytic analysis of the degradation of KGM by *Kg*ManA. The products generated by the degradation of KGM were analyzed by MALDI-TOF–MS. The degree of polymerization of konjac glucomannan oligosaccharides 2–9 is represented by the mass-to-charge ratios (*m/z*) (365 *m/z*, 527 *m/z*, 689 *m/z*, 851 *m/z*, 1013 *m/z*, 1175 *m/z*, 1337 *m/z* and 1499 *m/z*, respectively).

We utilized MALDI-TOF to examine the hydrolysis products of KGM yielded by *Kg*ManA. Our findings reveal that the hydrolysis products comprised various oligosaccharides with different degrees of polymerization (DPs) (Figure 4). In the positive mode, the mass-to-charge ratios (*m/z*) of the DPs to the products were predominantly calculated as 2 (365 *m/z*), 3 (527 *m/z*), 4 (689 *m/z*), 5 (851 *m/z*), 6 (1013 *m/z*), 7 (1175 *m/z*), 8 (1337 *m/z*) and 9 (1499 *m/z*) from the ion peak [DPx 2–12 + Na] + (x = 2–12) type. *Kg*ManA demonstrated a mannanase function based on the product distribution pattern and substrate specificity test. The recombinant mannanase *Kg*ManA is an endo-type enzyme that acts on the non-reducing end of the main chain of KGM. The mode of degradation of KGM by *Kg*ManA was similar to that of an enzyme derived from *Talaromyces cellulolyticus*, which degraded KGM into oligosaccharides with different degrees of polymerization (DP < 10) [35]. In summary, *Kg*ManA could be used as a mannanase for KGM degradation and to produce valuable KGMOS substrates.

### 3.5. In-Silico Analysis of the Mechanism of KgManA Degradation

**Figure 5:**
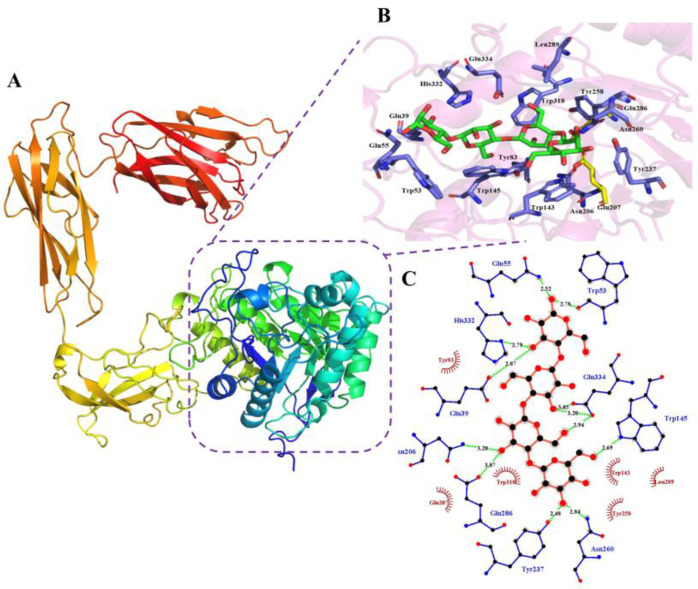
Overall structure of *Kg*ManA and interactions of the substrate in the active site of *Kg*ManA with mannotetrose. (**A**) Cartoon illustration of the complete *Kg*ManA function module. (**B**) Docked complex of *Kg*ManA. *Kg*ManA (purple), key catalytic amino acid (yellow), interactions with amino acid (blue). (**C**) Schematic diagram of key amino acids for *Kg*ManA–substrate interaction.

Based on its predicted structure, *Kg*ManA appears to be a protein with multiple domains, including an N-terminal GH5 catalytic domain module, a C-terminal CBM35 binding module, and two intermediate unknown functional domains (Fibronectin type 3 domain and myxosortase-dependent M36 family metallopeptidase domain) (Figure 5A). The overall structure of the *Kg*ManA N-terminal domain exhibits a typical (β/α)8 TIM-barrel architecture (Figure 5B). The active pocket of *Kg*ManA features a narrow and deep V-shaped groove that runs through the entire catalytic domain. The catalytic acid base and nucleophile (Glu207 and Glu286) are located at the top of the β-barrel. Four mannose residues are bound to the catalytic domain, occupying the −1 to +3 subsites. At the −1 subsite, Tyr237 and Asn260 form hydrogen bonds with the hydroxyl group on C-3, and Trp145 forms a hydrogen bond with the hydroxyl group on C-6, respectively, while Trp143, Tyr258, and Leu289 form hydrophobic interactions with glycosyl groups. At the +1 subsite, Asn206 and Glu286 form hydrogen bonds with the hydroxyl group on C-2, and Glu334 forms a hydrogen bond with the hydroxyl group on C-2. Additionally, Trp318 engages in a hydrophobic interaction with the glycosyl group. At the +2 subsite, Tyr83 interacts hydrophobically with the glycosyl group, whereas Glu334 forms a hydrogen bond with the hydroxyl group on C-2. And at the +3 subsite, Glu39 and His332 form hydrogen bonds with the hydroxyl group on C-2, while Gln55 and Trp53 form hydrogen bonds with the hydroxyl group on C-4 (Figure 5C).

## 4. Discussion

In this study, a novel mannanase, *Kg*ManA, from *K. grimontii* was cloned and expressed. The activity of this enzyme on the substrate KGM was studied, and its biological properties were characterized. Since *Kg*ManA has low homology with characterized mannanases, the study of this enzyme is of great value for exploring novel mannanases.

*Kg*ManA is highly sensitive to temperature, which may be related to the bacteria’s living environment. However, *Kg*ManA has outstanding pH stability and exhibits high activity across a wide pH range. This suggests that *Kg*ManA could be used for KGM degradation under specific pH conditions, and to create beneficial substrates for KGM degradation, this enzyme may be utilized in combination with an acid/alkali lysis strategy. 

The activity of *Kg*ManA towards the substrate KGM was different from those of the other typical β-1,4 mannanases. Most mannanases have activity against galactomannan, while *Kg*ManA does not possess this hydrolysis ability. For example, the activity of mannanase from *Aspergillus calidoustus* and *Alicyclobacillus* sp. strain A4 on locust bean gum exhibited enzyme activities of 669.7 U/mg and 370.4 U/mg, respectively [26,28]. The enzyme *Aa*ManA from *Alicyclobacillus acidocaldarius* exhibited a KGM activity of 724.4 U/mg [36]. These studies suggest that *Kg*ManA may possess different catalytic properties than typical β-mannanase. Similar to *Kg*ManA, the enzyme *Ba*Man113A was derived from *Bacillus* sp. N16-5, and had an activity of only 1.040 ± 0.079 U/mg and 0.937 ± 0.028 U/mg on KGM and locust bean gum, respectively. However, it was able to efficiently degrade natural polysaccharides into oligosaccharides [37]. Therefore, based on the product analysis test of *Kg*ManA, we speculate that *Kg*ManA may also be more inclined to degrade KGM into oligosaccharides rather than monosaccharides. This indicates that this mannanase has the potential to be applied in the production of KGMOS.

Moreover, substrate-specific studies have revealed the high specificity of *Kg*ManA. A variety of polysaccharide substrates with different structures were used to evaluate the specificity of *Kg*ManA. Among them, KGM is the target substrate of *Kg*ManA, and β-1,4-D-mannan is used to verify its mannanase activity. In addition, curdlan is a linear 1,3-β-glucan [38,39], CMC is a 1,4-β-glucan containing side chains with carboxymethyl groups [39], and zymosan A is a linear 1,3-β-glucan with 30-residue long branches [40]. Some polysaccharides derived from plants were also used to assess the specificity of *Kg*ManA towards KGM. Locust bean gum (LBG), tara gum (TG), guar gum (GG), sesbania gum (SG), and fenugreek gum (FG) are galactomannans with varying amounts of galactose side chains [41]. When the mannose to galactose ratio (M/G) was used, the M/G values corresponding to these polysaccharides were 4:1 (LBG), 3:1 (TG), 2:1 (GG), 2:1 (SG), and 1:1 (FG), respectively [42,43,44,45,46]. According to the results, *Kg*ManA appears to have no activity on polysaccharides without mannose (such as CMC, curdlan, and zymosan A) in the main chain. It only hydrolyzes polysaccharides that contain mannose residues in their main chain. Unexpectedly, *Kg*ManA could hydrolyze KGM with acetyl side chains, but was unable to hydrolyze mannan with galactose side chains (LBG, TG, GG, SG, and FG). We suppose there could be two causes. Firstly, *Kg*ManA has poor hydrolytic activity and cannot produce activity on various viscous polysaccharides. On the other hand, the acetyl side chain of KGM may structurally support the catalytic activity of this enzyme. Therefore, *Kg*ManA acts more actively on KGM than on other natural polysaccharides. Secondly, galactose on the polysaccharide side chain may competitively inhibit the activity of mannanase *Kg*ManA. When the polysaccharide’s side chain contains galactose, the enzyme is competitively bound, thus preventing it from reacting with the polysaccharide’s main chain. However, *Kg*ManA seems to have a weak activity against fenugreek gum, which we assume might be related to the amount of its side chains. Galactomannan, with a higher number of galactose side chains, has a reduced number of intrachain hydrogen bonds formed by mannose units. This, in turn, facilitates the exposure of mannose units and their glycosidic bonds [41]. Among all the tested natural galactomannans, FG has the highest M/G value, fewer intrachain hydrogen bonds, and higher solubility. As a result, its glycosidic bonds are more easily exposed to the dissolved *Kg*ManA, and may be partially degraded. However, the negligible enzyme activity does not support the idea that *Kg*ManA could effectively degrade mannan with galactose side chains. In summary, *Kg*ManA can be used to specifically break down KGM-like polysaccharides in mixed substrates, resulting in the production of oligosaccharides without galactose side chains.

*Kg*ManA is a novel KGM-degrading enzyme that is capable of degrading KGM over a wide pH range and producing typical degradation products of KGM mannanase. This conclusion is based on pH stability assays, substrate specificity results, and product analysis. The modification of *Kg*ManA activity will serve as the foundation for its expanded and future applications. Additionally, further research on *Kg*ManA will help improve the specificity and pH stability of other KGM-degrading enzymes.

## 5. Conclusions

In this study, *Kg*ManA was cloned from *K. grimontii* DICP B2-5 and successfully expressed heterologously in *E. coli* BL21 (DE3). Based on the bioinformatic and phylogenetic analysis, *Kg*ManA is a novel mannanase with less than 18.33% structural similarity to other enzymes, and is distinct from traditional mannanases. According to the results of activity determination and substrate specificity analyses, *Kg*ManA has an optimum temperature of 40 °C and an optimum pH of 6.0. Furthermore, this enzyme specifically hydrolyzes mannan without galactose in the side chain, and could degrade KGM into oligosaccharides with a degree of DP ranging from two to nine. Additionally, structural simulation and substrate molecular docking were performed here for the first time. All the results show that *Kg*ManA is promising for use in producing KGMOS, and it has the potential to contribute to ongoing research, which can aid in the development of *K. grimontii*-derived mannanase.

## Figures and Tables

**Table 1:** Purification of *Kg*ManA.

Purification Process	Total Protein (mg)	Total Activity (U)	Specific Activity (U/mg)	Purification Factor	Yield (%)
Crude enzyme	2234.93	502.78	0.22	1.00	100
NTA-Ni resin	9.60	2.33	0.24	1.08	0.46

**Table 2:** Substrate specificity of *Kg*ManA.

Substrate	Hydrolytic Activity (U/mg)	Relative Activity (%)
KGM	0.43 ± 0.04 *	34.40 ± 3.20
β-1,4-D-mannan	1.25 ± 0.01 *	100.00 ± 0.80
carboxymethyl cellulose	NA ^1^	NA ^1^
curdlan	NA ^1^	NA ^1^
locust bean gum	NA ^1^	NA ^1^
fenugreek gum	0.04 ± 0.01 *	3.20 ± 0.80
guar gum	NA ^1^	NA ^1^
Sesbania gum	NA ^1^	NA ^1^
zymosan A	NA ^1^	NA ^1^
tara gum	NA ^1^	NA ^1^

^1^ NA, the activity of *Kg*ManA hydrolysis of the polysaccharide was not detected. *, there are significant differences between these groups based on one-way ANOVA analysis, *p* < 0.01.

**Table 3:** Enzyme kinetic constant of *Kg*ManA.

Enzyme Kinetic Parameters	Value
K_m_	9.04 ± 1.11 mg/mL
V_max_	0.77 ± 0.18 μmol/(min·mg)
K_cat_	1.53 ± 0.36 min^−1^
K_cat_/K_m_	0.17 ± 0.03 mg^−1^·mL^−1^·min^−1^

## Data Availability

Not applicable.

## Supplementary Materials

The following supporting information can be downloaded at: https://www.mdpi.com/article/10.3390/bioengineering10101230/s1, Figure S1: Electrophoretic detection of the amplified product of *Kg*ManA. Lane M: DNA marker; Lane 1: PCR products of KgManA. Figure S2: SDS-PAGE of the purified *Kg*ManA. Lane 1: uninduced culture; Lane M: protein marker; Lane 2: cure enzyme; Lane 3: protein flow through Ni–NTA resin; Lane 4: washing by binding buffer containing 20 mM of imidazole; Lane 5: washing by wash buffer contained 80 mM of imidazole; lane 6: washing by elution buffer contained 250 mM of imidazole. S3: Protein sequence of *Kg*ManA in fasta-format.
