# Supplementary material for: Identification and Characterization of a Novel Mannanase from Klebsiella grimontii
Source: Bioengineering (Basel). 2023 Oct 21;10(10):1230. doi: 10.3390/bioengineering10101230 (PMC10604067; doi:10.3390/bioengineering10101230)
Supplement: Supplementary file 1 [file bioengineering-10-01230-s001.zip › bioengineering-2647159-supplementary.pdf]

bioengineering

Identification and Characterization of a Novel Mannanase from *Klebsiella grimontii*

Changzheng Chen [1,2,†], Kuikui Li [1,*,†], Tang Li [1], Junyan Li [1], Qishun Liu [1,3] and Heng Yin [1,2,*]

1 Dalian Engineering Research Center for Carbohydrate Agricultural Preparations, Dalian Technology Innovation Center for Green Agriculture, Liaoning Provincial Key Laboratory of Carbohydrates, Dalian Institute of Chemical Physics, Chinese Academy of Sciences, Dalian 116023, China
2 University of Chinese Academy of Sciences, Beijing 100190, China
3 Key Laboratory of Se-enriched Products Development and Quality Control, Ministry of Agriculture and Rural Affairs, National-Local Joint Engineering Laboratory of Se-enriched Food Development, Ankang 725000, China
* Correspondence: lkk@dicp.ac.cn (K.L.); yinheng@dicp.ac.cn (H.Y.)
† These authors contributed equally to this work.

Supplementary Materials

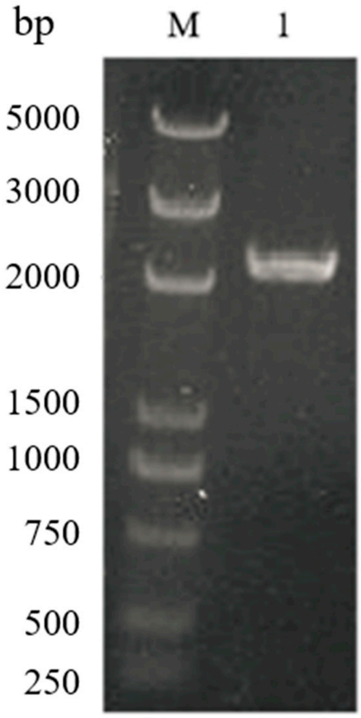

Figure S1. Electrophoretic detection of the amplified product of *Kg*ManA. Lane M: DNA marker; Lane 1: PCR products of *Kg*ManA.

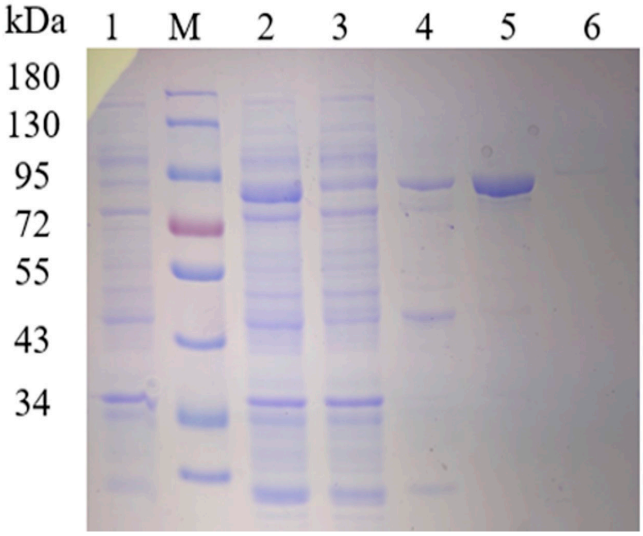

Figure S2. SDS-PAGE of the purified *Kg*ManA. Lane 1: uninduced culture; Lane M: protein marker; Lane 2: cure enzyme; Lane 3: protein flow through Ni–NTA resin; Lane 4: washing by binding buffer containing 20 mM of imidazole; Lane 5: washing by wash buffer contained 80 mM of imidazole; lane 6: washing by elution buffer contained 250 mM of imidazole.

Protein sequence of KgManA in fasta-format

>*Kg*ManA (716 aa)
MAEQSHFEHFITRDGATLKDGDKVFRFAGIHAPELHRIEDDARGTCKADTRGWGQYFRWP-
TAEEQENWIKAMVQTGARAQRVYVLSVQQTFDEACGRETHILAPETTDGMPRLNEKAMRVYDNMIAEADKQGLRLILPFIDHW
WWWGGREQLAAFYHEKPEDFYRTDSKTFKVYLDVIRQVITRTNSVTGRPYFDEKA-
IMAWETGNELEDTNAAFLQQTAAWIKKWAPHQLVVDGTYKKINGFALNDPNVDIVSNHYYTNADNNHPDQVKKDLTAAAG
KKVYMVGEFGLLDAQQLNAIMQSIVHSEVNGAQAAGGLIWGFRGHRHDGGFY-
WHKESTGHYSYHLPGFPMEGKANQEMEVVDLVRTAAAQMNGQENAPPLPKPDAPTLRATDSPFAINWLGAAVGRAYDVERA
DSASGPWKVVGRDISDGVNEWNPQTMDLFRDDYRSLQLGNTYYYRVIAKNESGSSAPSNVIS-
VKHTQANQAPVVALAETLTTSQDQGVQLSASWRDDGLPDRDVKVNWSNGGSAQAHFCATDKAETRAWFSAPGEYALTFSAD
DGLLKSSKTVKVTVTEAVGKVPADYCRFGGGVLHVTEGKIEAAKSEKDAL-
TIDEDGFLGPFANDGDKVSWQVSAPWAGKYLLRVTFSGKWGGKKNSFIVNGGAPIAVEFPQTDEQGQQQLVPVELKAGDNRIDF
GKFAGDWGYMFIKSIEEGAELEHHHHHH
